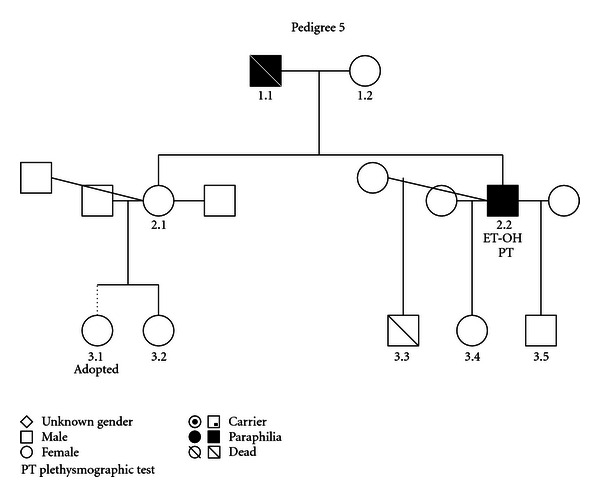# Familial Paraphilia: A Pilot Study with the Construction of Genograms

**DOI:** 10.5402/2012/692813

**Published:** 2012-03-04

**Authors:** Alain Labelle, Dominique Bourget, John M. W. Bradford, Martin Alda, Pierre Tessier

**Affiliations:** ^1^Schizophrenia Program, Royal Ottawa Mental Health Centre, University of Ottawa, Ottawa, ON, Canada K1Z 7K4; ^2^Integrated Forensic Program, Royal Ottawa Mental Health Centre, University of Ottawa, Ottawa, ON, Canada K1Z 7K4; ^3^Division of Forensic Psychiatry, University of Ottawa, Ottawa, ON, Canada K1Z 7K4; ^4^Dalhousie University, Halifax, NS, Canada B3H 3J5; ^5^Mood Disorders Program, Royal Ottawa Mental Health Centre, University of Ottawa, Canada K1Z 7K4

## Abstract

Biological factors are likely predisposing and modulating elements in sexually deviant behavior. The observation that paraphilic behavior tends to cluster in some families is intriguing and potentially raises questions as to whether shared genetic factors may play a role in the transmission of paraphilia. This pilot study introduces five families in which we found presence of paraphilia over generations. We constructed genograms on the basis of a standardized family history. Results document the aggregation of sexual deviations within the sample of families and support a clinical/phenomenological heterogeneity of sexual deviation. The concept of paraphilia in relation to phenotypic expressions and the likelihood of a spectrum of related disorders must be clarified before conclusions can be reached as to family aggregation of paraphilia based on biological factors.

## 1. Introduction

Paraphilias are classified in the *Diagnostic and Statistical Manual of Mental Disorders *(DSM-IV) [1] as sexual disorders characterized by intense, recurrent sexual fantasies, thoughts, and/or behaviors. Diagnostic criteria for these disorders require either marked subjective distress or interpersonal difficulty. Despite operational categorization as psychiatric disorders, paraphilias have not been established as classical major mental illnesses such as schizophrenia or affective disorder and have often been equated with antisocial behavior. Although this argument has been raised, the DSM-IV, in effect, has characterized the paraphilias as Axis I Disorders involving a fundamental aberration in sexual makeup and phenomenology. In that sense, simply behaving in an antisocial fashion by engaging in sexually improper behavior does not necessarily constitute a sufficient basis for diagnosing a paraphilic disorder. Of the various paraphilias, the most common forms encountered in clinical practice involve pedophilia, voyeurism, and exhibitionism [1]. Individuals suffering from pedophilia, voyeurism, or exhibitionism represent most apprehended sexual offenders [1]. Several forensic psychiatric programs run specialized clinics to address the need for risk assessment of these individuals and further, to provide comprehensive assessments and treatments. In this context, it would be helpful to gain a better understanding of the etiology of paraphilia as it may bear a direct impact on treatment and counseling. 

 While the etiology of paraphilias remains largely unknown, various theories have been put forth to account for the occurrence of these sexual disorders. According to social learning theory, most aspects of human sexual behavior, including deviant sexual behavior, are primarily modulated by learning and modeling processes resulting from social and familial influences [2, 3]. A number of models have proposed that negative experiences during childhood, such as parental violence or dysfunctional parent-child relationships, are important precursors to sexual offending with or without paraphilia [4–6]. Several studies aimed at identifying developmental psychopathology associated with specific paraphilias have suggested that an individual's own experience of childhood sexual abuse is a risk factor for later pedophilic behavior [7–13]. However, in a review of the literature on histories of child abuse in sex offenders, Garland and Dougher [14] noted that there is much variance in reported rates of childhood sexual abuse in sex offenders of children (0%–57%), other sex offenders (8%–57%), nonsexual offenders (10%–47%), and nonoffenders (3%–16%). These authors concluded that sexual victimization is neither a necessary nor a sufficient precursor of pedophilic interest in adulthood. Another notable review of the literature also found no evidence of a specific relationship between childhood sexual abuse and later abusing in samples of sex offenders, nonsexual offenders, and nonoffenders [15]. Despite reports that suggest an elevated rate of a history of sexual victimization in pedophile offenders [9, 16], a causal relationship between childhood abuse and adult pedophilia has not been clearly established.

 While psychological factors may well influence whether or not an individual will act on his or her sexual impulses, it is likely that biological factors are predisposing and modulating elements to aberrant sexual behavior. Physiological assessments have shown that rapists and violent pedophilic males have a distinct pattern of sexual arousal compared to control groups [17–19]. The effectiveness of hormonal organic treatments for male sexual offenders further supports the role of biological factors in the modulation of sexually deviant behaviors [20].

 A number of studies have examined other biological correlates in paraphilic sexual disorders in an attempt to determine causal factors. Evidence of endocrine and brain pathology has been found in some individuals with paraphilia [21–27]. Two independent investigations of endocrinological function in pedophilia found that pedophilic patients had elevated responses of luteinizing hormone (LH) to the infusion of luteinizing hormone-releasing hormone [21] or gonadotropin-releasing hormone (GRH) [22]. Casanova et al. [27] suggested that the marked LH response to GRH in individuals with pedophilia may be consistent with hippocampal pathology, given the sensitivity of hippocampal receptors to gonadal steroids. These investigators found pyramidal cell hippocampal atrophy when they examined the brains of two men diagnosed with paraphilia and schizophrenia or bipolar disorder. These authors note the unlikelihood that a comorbid psychiatric diagnosis or medication use could account for their findings, as the neuropathological changes were not evident in 18 control patients diagnosed with schizophrenia and treated with neuroleptic medication. Evidence of frontal and temporal abnormalities has also been found in individuals with pedophilia and/or other paraphilias [24, 28–30].

 As additional evidence that paraphilia may have a biological basis, two recent studies have reported an association between handedness and erotic age preference. Cantor et al. [31, 32] assessed 404 men who had clinically significant sexual behaviors or interests, nearly half of whom had committed a sexual offense against victim(s) aged 11 or under. These investigators found that patients' right-handedness was negatively correlated with their phallometric responses to erotic stimuli depicting prepubescent children, and positively correlated with stimuli depicting adults. As non-right-handedness occurs more frequently in people with various neurological disorders than in the general adult population [33–35], Cantor et al. [32] suggest that elevated levels of non-right-handedness in pedophiles indicates a relationship between pedophilia and brain organization, similar to that of other major neurological conditions.

 Paraphilias are associated with elevated rates of psychiatric comorbidity, including affective disorders, anxiety, and impulse control disorders, substance abuse disorders, and personality disorders [36, 37]. Recent developments in molecular biology and linkage analysis have contributed to research for causes and pathogenesis of psychiatric illnesses. Evidence for genetic transmission of major illnesses such as schizophrenia, affective disorder, and alcoholism has been reported in a number of studies [38–44]. While few studies have investigated the genetics of paraphilia, Schiavi et al. [45] found significantly more aberrant sexual activity and fantasy in XYY men compared to XXY men and those in a control group. Briken et al. [46] found that the rate of the XYY chromosome abnormality was much higher in men who had committed sexually motivated homicide, compared to the general population. Sexually sadistic behavior was also diagnosed in the XYY perpetrators.

 Familial transmission of pedophilia was questioned in a previous study that compared families with pedophilia and families with a nonpedophilic paraphilia [47]. Gaffney et al. [47] found that over 18 percent of all of the families had first degree relatives with a sexual deviancy. Family members of the patients with pedophilia also had pedophilia, with no other paraphilia evident in the families. In the families with a nonpedophilic paraphilia, sexual deviancy rarely involved children. These investigators suggested that pedophilia is familial and specific and noted the need for further studies to clarify the manner of transmission.

 Generally speaking, a systematic collection of family pedigree data may yield evidence that an illness is likely to have a genetic basis and provide support for further research with more sophisticated techniques [38, 40, 41, 48]. However, several problems limit the interpretation of pedigree data. Selection of appropriate families, accuracy of diagnostic methods, and diagnostic limitations remain significant obstacles [40]. Difficulties associated with genetic principles such as complex gene-environment interactions, heterogeneity, incomplete penetrance/manifestation in predisposed individuals, and variability of phenotypic expression further complicate research [38, 40, 41]. Suitable families for adoption studies as well as twin studies on paraphilia are not likely to be conducted on a large scale.

 We present the pedigrees of five families with familial aggregation of paraphilias. Although several limitations are found in the interpretation of the data, the identification of families with multiple members affected with paraphilia can be a starting point in assessing the feasibility of larger studies on familial paraphilia to help us gain some understanding on the influence of biological and possibly genetic factors on the expression of sexually deviant behavior.

## 2. Method

This pilot study was undertaken in a university-affiliated forensic psychiatric program Sexual Behaviors Clinic after receiving ethics approval by the institutional ethics board. The Sexual Behaviors Clinic offered services to a population of patients who were all referred through either a medical source or a legal source for assessment and treatment of a sexual paraphilia. Target subjects included in the study were all patients duly registered in the clinic in whom a diagnosis of paraphilia had been made following a comprehensive assessment which included a detailed psychiatric history, review of antecedents, self-report questionnaires, and standard penile plethysmographic testing. As part of the usual history taking, most patients were routinely questioned about their family history. Notwithstanding any result obtained on the questionnaires or plethysmographic test, the diagnosis of paraphilia was based on the subject clinically meeting DSM-IV criteria for one of the paraphilias. [1] Inter-rater agreement on the diagnosis of paraphilia in each case was provided by two forensic psychiatrists trained in the area of sexual disorders (paraphilias). In this manner, 14 families in which a familial paraphilic pattern might be present were identified out of approximately 200 new referrals (7%), based on reported antecedent family history of the subject. A familial paraphilic pattern was defined by the presence of at least two [2] first- or second-degree relatives who had already been diagnosed with a sexual paraphilic disorder through contacts with the Sexual Behaviors Clinic. Five out of the 14 families (36% of sub-sample) agreed to participate in the study. Key informants, that is, the patient himself or a knowledgeable family member with the patient's permission, were identified in each family. Explanations of the Canadian legislation on the mandatory report of child abuse were given to subjects and family key persons. After a complete description of the study was given to the subjects, written informed consents were obtained. Detailed interviews were conducted using the Family History-Research Diagnostic Criteria (FH-RDC) [49]. While the FH-RDC does not screen for sexual disorders, it provides a framework to query the presence or absence of other major psychiatric disorders in relatives. In certain individuals, the presence of some disorders (such as psychosis or an organic condition) may cause them to engage in aberrant sexual behaviors of a nonparaphilic nature. The presence of a major psychiatric disorder was not construed as an exclusionary criterion as such but it could have represented a confounding factor worth documenting. In addition, specific inquiries were made to the key informants in relation to a history of abnormal or sexually disordered behavior in family members, based on the DSM-IV diagnostic criteria under the Sexual Disorders category, specific to the paraphilias [1]. As key informants would not necessarily know whether a given family member had experienced “*recurrent, intense sexually arousing fantasies or sexual urges* (…),” the inquiry focused on the behavior component of criterion A. For instance, a person would be classified as meeting the criteria for pedophilia if “*over a period of at least 6 months …behaviors involving sexual activity with a prepubescent child or children*” and “the *person has acted on these sexual urges*”. We applied a similar approach for sexual sadism, the other type of paraphilia encountered in the sample. It is acknowledged that this “reductionist” approach to the diagnostic category, which was reasonable under the circumstances to ensure reliability of diagnosis in the class, would likely lead to an underestimation of the condition. This being said, only those cases that truly satisfied the DSM criteria for paraphilia were classified as such. Other cases in which the sexual behavior or sexual habits were “unconventional” or raised concerns (e.g., hypersexuality, multiple mating (multiple sexual partners), etc.) were identified and noted accordingly in the pedigrees. Collateral information was also collected regarding subjects' history. When reviewing history of physical problems, the conditions that stood out were recorded for the interest they may present for future studies, including deafness, mutism, cerebral palsy, and epilepsy.

 Genograms were constructed on the basis of a standardized family history, using the standardized model proposed by McGoldrick et al. [50].

## 3. Results

Results of our investigation are reported below. Relevant details pertaining to the presence of paraphilia, comorbid psychiatric conditions, and medical conditions are provided on the genograms of the five families. Individuals who underwent plethysmographic testing are among the individuals who were specifically assessed in the clinic and provided first-hand information to the investigators (key informants). They were identified in the genograms (PT).

### 3.1. (See Figure 1)

In this family, we ascertained the presence of a paraphilic disorder by a direct clinical examination of individuals 2.1, 2.4, and 2.5. All affected family members from Pedigree 1 suffered from pedophilia. While two presented with heterosexual pedophilia (2.1 and 2.5), one had homosexual pedophilia (2.4). Two displayed sexual sadism (2.4 and 2.5). Individual 2.5 had murdered a 4-year-old girl. Other behavioral manifestations in the family included alcohol abuse (1.1 and 2.1), drug abuse and diagnosis of conduct disorder in childhood (2.3), antisocial personality disorder, and borderline intelligence (2.4). Individual 2.7 had cerebral palsy.

### 3.2. (See Figure 2)

 The occurrence of heterosexual pedophilia runs over four generations in males of this family (1.1, 2.1, 3.2, 3.4, 3.5, 3.6, 4.5, 4.9, and 4.14). Individual 4.4 suffered from an auditory problem while individual 4.12 (a female) had cerebral palsy.

### 3.3. (See Figure 3)

 Affected members of this pedigree manifested homosexual pedophilia with sadism (3.1, 3.2, 3.5, and 4.17). All four were known to the criminal justice for repeated sexual offences. Individual 4.11 was involved in prostitution. Many individuals (3.1, 3.3, 3.4, 3.8, and 3.12) in the third generation suffered from deafness and muteness, while individuals 3.11 and 4.11 had epilepsy. Little information is known about the second generation except that four individuals were deaf and mute. One female individual (3.12) with a history of multiple mating has an affected descendant (4.17). We consider the possibility that this female may carry a genetic predisposition (a “carrier”).

### 3.4. (See Figure 4)

 A heterogeneous spectrum including heterosexual pedophilia (2.1, 2.4, 2.13, 3.1, 3.9, 3.12, and 3.13) and adult sexual sadism (3.3, 3.11, and 3.12) occurs over two generations. Poor control over impulsive behavior with aggressive outbursts and physical violence was present in individuals 2.2, 2.12, 3.3, and 3.11. Three individuals (2.1, 2.4, and 2.12) were blind. A history of multiple marriages/affairs is present in one affected member (2.13) and his sibling (2.8). We consider the possibility that individuals 2.3 and 2.8 may be “carriers” of a genetic predisposition.

### 3.5. (See Figure 5)

 Affected members suffered from pedophilia. Individual 1.1 was sexually attracted to females and individual 2.2 to males. Individual 2.1 married three times. While she adopted child 3.1, she gave birth to a muscular disabled child (3.2). Individual 2.2 abused alcohol.

## 4. Discussion

 Although the etiology and pathophysiology of paraphilias remain unclear, a review of the literature suggests the influence of psychosocial as well as neurobiological factors in the development of deviant sexual behavior. Paraphilias are behaviors likely resulting from an interaction between genetic and psychosocial factors as well as additional factors such as impaired inhibition, for instance due to substance abuse or decreased intelligence. Little research has investigated familial aggregation in paraphilias, and to our knowledge no previous research has presented genogram data on paraphilia.

 We have illustrated the presence of a family aggregation of paraphilia in selected families. In these families the rates of paraphilia are considerably higher than what would be expected based on population prevalence data. In the genograms presented, there are 26 affected males and one female. Although there could be initial speculation as to Y-chromosome transmission, this does not appear to hold true, as there is one affected female. Moreover, of three hypothetical “carriers” of a genetic abnormality (individual 3.12 in Pedigree 3 and individuals 2.3 and 2.8 in Pedigree 4), one is a female. Many instances of vertical transmission point against uniformly recessive inheritance (as paraphilia is not frequently diagnosed in the general population). The presence of several “carriers” might be an indicator that the genetic abnormality is not fully penetrant and that environmental factors may modify the phenotypic expression. It will be necessary to investigate further as to whether there are other (nonsexual) syndromes that are more frequently encountered in families with paraphilia.

 Multiple types of paraphilia were evident in the genograms presented, with some of the subjects presenting with heterosexual pedophilia, homosexual pedophilia, and/or sexual sadism. While this illustrates the phenotypic spectra of paraphilia present in these families, it is important to note that most of the affected family members suffered from pedophilia (homosexual or heterosexual), lending further support to the findings of Gaffney et al. [47] and suggestion of specificity in the familial transmission of paraphilia. The theoretical implications of cerebral palsy, deafness, mutism, and epilepsy in the sample remain unexplained in the present state of knowledge. Cryan et al. [51] presented a pair of monozygotic twins concordant for both OCD and paraphilia and suggested there may be an organic basis to these conditions, quoting case reports of deviant sexual behavior associated with brain pathologies.

 We are aware that the ascertainment or selection procedure has led to a higher representation of affected relatives in this sample compared to the overall population seen in the clinic. However, it is likely that the occurrence of paraphilia within the sample was underreported due to methodological limitations such as the investigation method (FH-RDC and direct questioning of sexual behavior) the legislation warnings given to the informants that potentially acted as a deterrent, as well as the nature of the disorder and area of secrecy surrounding it. The question of the diagnosis can be a great source of variability in this type of study, and represents a significant challenge as pointed out by Kety in the initial stages of research in the genetics of schizophrenia [52]. 

In an attempt to overcome this, we used strict criteria as those elaborated in the DSM, knowing this could lead to underrepresentation of the disorder. The method of using key informants may also have a low sensitivity to detect noteworthy features of a possible paraphilia spectrum such as hypersexuality and intermittent paraphilic interests associated with the use of substances, for example.

 Difficulties inherent to diagnostic methods are particularly relevant to the sexual disorders, where one finds considerable heterogeneity and overlap with several varieties of deviant impulses. Nonexclusivity of deviant sexual interests and variable age of onset of paraphilia pose further problems in correctly identifying all affected members at any given time. Age-dependent penetrance could affect the expression of the disorder at the time of collecting data. The specificity of a particular disorder may be more difficult to establish when confronted mostly with behavioral tendencies, as is often encountered in clinical practice. For instance, hypersexual behavior frequently coexists with paraphilias [53–55] and has been described as a paraphilia-related disorder [53, 56]. Hypersexuality might be part of a spectrum of related diseases. Consideration should be given to the idea that different manifestations of behavior may ultimately have a common denominator. Brunner et al. [57] described a large family in which several males with borderline mental retardation exhibited prominent behavioral disturbance, including a tendency toward aggressive outbursts and other impulsive behaviors (i.e., arson, attempted rape, and exhibitionism). Abnormal behavior in five of the males was associated with a point mutation in the structural gene for monoamine oxydase A (MAOA), indicating that MAOA deficiency was associated with a behavioral phenotype that included disturbed regulation of impulsive aggression. It is possible that multiple mating/affairs represent a similar phenomenon, and these occur in both sexes. Whether relatives with multiple mating/affairs could be considered carriers of the genetic predisposition to paraphilia remains a concept to be explored in light of the association between paraphilia and hypersexual behavior. Analysis of the mode of inheritance, if the hypothesis holds true, will be complicated by lack of epidemiological data on paraphilia, with little information about population prevalence. While paraphilia is rarely diagnosed in clinical facilities, the prevalence in the community is believed to be far higher [1]. Furthermore, selection of families with multiple affected cases leads to an ascertainment bias, but this bias is well defined and can be corrected by statistical means.

 We considered the possibility of a genetic transmission for paraphilia and constructed genograms on the basis of a standardized family history. On further analysis, it was not possible to demonstrate any simple mechanism of genetic inheritance in this sample. This may have been at least in part due to the heterogeneity of the sexual behaviors manifested within the five families. Our sample of family aggregation of paraphilia supports a hypothesis of psychosocial familial transmission and/or possible genetic transmission. Although this pilot study cannot differentiate between the two possible causes of familial aggregation, it is nevertheless a constructive step in suggesting a pattern of familial aggregation in relatives of individuals with paraphilia, particularly given the lack of family and other studies. If our finding of family aggregation of paraphilia is secondary to genetic or intrafamilial environmental factors, the issue of phenotypic expression remains unclear.

 This pilot study does not provide an answer to the presumptive biological roots of paraphilia; however the finding that the disorder tends to cluster in some families could potentially support a hypothesis that it represents the expression of shared genetic factors and lend support to further studies in this area. Future studies could look at a more homogeneous group manifesting a single paraphilic disorder, such as same gender pedophilia of the exclusive type. The concept of paraphilia in relation to phenotypic expressions and the likelihood of a spectrum of related disorders need further clarification before conclusions can be reached on family aggregation of paraphilia based on biological factors. A controlled study with replication of pedigree data on a larger scale, focusing as much as possible on high-density pedigrees, is indicated.

## Figures and Tables

**Figure 1:**
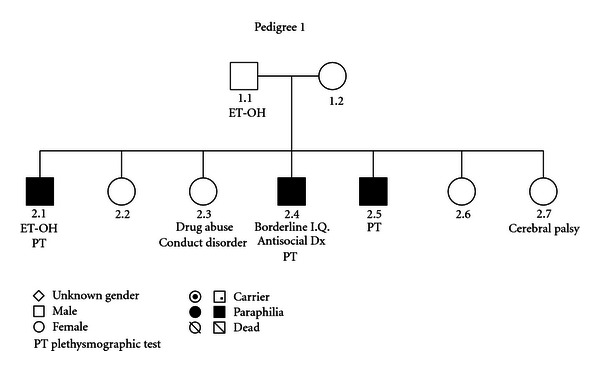


**Figure 2:**
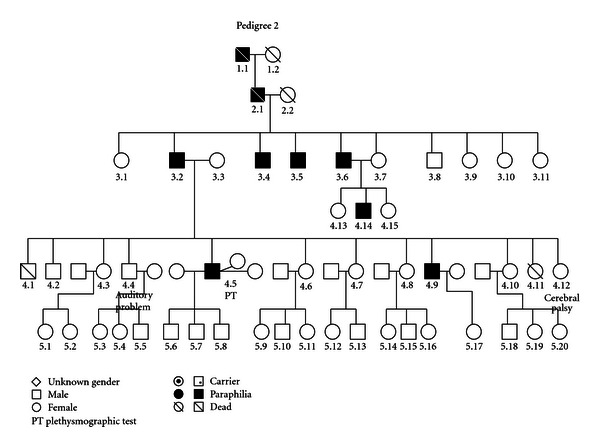


**Figure 3:**
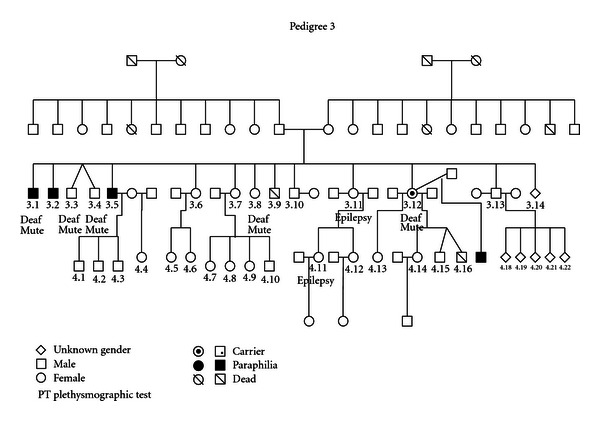


**Figure 4:**
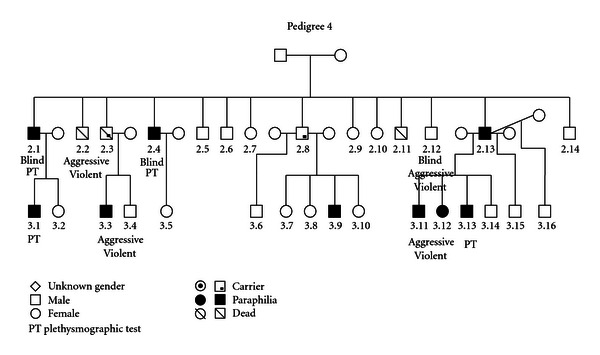


**Figure 5:**